# Experimental and Clinical Aspects of Sevoflurane Preconditioning and Postconditioning to Alleviate Hepatic Ischemia-Reperfusion Injury: A Scoping Review

**DOI:** 10.3390/ijms24032340

**Published:** 2023-01-25

**Authors:** Loïc Benoit, Audrey Dieu, Maxime Foguenne, Eliano Bonaccorsi-Riani

**Affiliations:** 1 Department of Anesthesiology, Cliniques Universitaires Saint-Luc, 1200 Brussels, Belgium; 2 Abdominal Transplant Unit, Cliniques Universitaires Saint-Luc, 1200 Brussels, Belgium; 3 Pôle de Chirurgie Expérimentale et Transplantation-Institut de Recherche Expérimentale et Clinique, Université Catholique de Louvain, 1200 Brussels, Belgium

**Keywords:** ischemia reperfusion injury, sevoflurane, liver transplantation, anesthesia

## Abstract

Ischemia-reperfusion injury (IRI) is an inflammatory process inherent in organ transplantation procedures. It is associated with tissue damage and, depending on its intensity, can impact early graft function. In liver transplantation (LT), strategies to alleviate IRI are essential in order to increase the use of extended criteria donor (ECD) grafts, which are more susceptible to IRI, as well as to improve postoperative graft and patient outcomes. Sevoflurane, a commonly used volatile anesthetic, has been shown to reduce IRI. This scoping review aims to give a comprehensive overview of the existing experimental and clinical data regarding the potential benefits of sevoflurane for hepatic IRI (HIRI) and to identify any gaps in knowledge to guide further research. We searched Medline and Embase for relevant articles. A total of 380 articles were identified, 45 of which were included in this review. In most experimental studies, the use of sevoflurane was associated with a significant decrease in biomarkers of acute liver damage and oxidative stress. Administration of sevoflurane before hepatic ischemia (preconditioning) or after reperfusion (postconditioning) appears to be protective. However, in the clinical setting, results are conflicting. While some studies showed a reduction of postoperative markers of liver injury, the benefit of sevoflurane on clinical outcomes and graft survival remains unclear. Further prospective clinical trials remain necessary to assess the clinical relevance of the use of sevoflurane as a protective factor against HIRI.

## 1. Introduction

Ischemia-reperfusion injury (IRI) refers to a pathophysiological process caused by organ or tissue ischemia and subsequent resumption of blood flow and tissue reoxygenation. 

It involves numerous complex pathophysiological mechanisms that can be summarized in two stages: ischemia and reperfusion. During ischemia, alterations in cell metabolism caused by energy depletion play a major part in subsequent cell and tissue damage. Ischemic injury is further associated with increased cytokine production and the expression of endothelial and parenchymal adhesion molecules. At the reperfusion stage, oxidative stress, microvascular dysfunction and a further increase in inflammatory mediators play a central role [1,2].

Hepatic IRI (HIRI) can occur in various clinical situations, such as hemorrhagic shock or liver resection surgery, in which inflow occlusion (Pringle’s maneuver) is applied in order to reduce blood loss. It is intrinsically linked to solid organ transplantation procedures, where grafts are exposed to a long succession of ischemic injuries before reperfusion in the recipient. The resulting damage can have a significant impact on graft recovery, but also trigger a cascade of systemic reactions leading to multiple organ dysfunction after transplantation [3,4]. 

To this day, liver transplantation (LT) remains the only curative treatment for end-stage liver disease. Improvements in surgical techniques, immunosuppressive therapies, preservation strategies and perioperative management have been significant over the past decades. These improvements have led to an increasing demand for liver grafts and a growing disparity between demand and supply. Thus, a rising number of organs are now being procured from extended criteria donors (ECDs), such as obese or elderly donors and donors after circulatory death (DCD). These organs are known to be more susceptible to IRI [5]. In light of these problems, the mitigation of IRI is essential in order to increase the use of organs from ECDs, as well as to improve postoperative outcomes after LT. 

Multiple surgical and non-surgical strategies have been proposed to alleviate HIRI. These IRI alleviation approaches are usually referred to with the term “conditioning”. Depending on the time of application, the terms “preconditioning” (before onset of ischemia), “conditioning” (during ischemia) or “postconditioning” (upon reperfusion) are generally used. Preconditioning with short periods of ischemia (ischemic preconditioning, IPC) was first described in an animal model of myocardial IRI in 1986 and was found to significantly reduce infarct size [6]. Numerous clinical trials later demonstrated a benefit of IPC and intermittent clamping (IC) on HIRI [7,8,9], and both strategies became popular in liver surgery. However, the clinical benefit of IPC still remains controversial [10,11]. 

As surgical strategies require surgical manipulation of the organ and usually prolong the duration of surgery, pharmacological approaches seem to be more easily applicable and less invasive alternatives. Numerous molecules have been studied and shown to reduce IRI, including erythropoietin, ulinastatin, methylprednisolone, N-acetylcysteine or volatile anesthetics (VAs), such as sevoflurane [1,2].

Sevoflurane is a widely used, non-pungent VA known for its fast onset and offset and the hemodynamic stability it provides. The chemically related anesthetic halothane was found to be a rare cause of liver injury and has now been largely replaced by the newer halogenated anesthetics isoflurane, desflurane and sevoflurane. Halothane hepatotoxicity is thought to be caused by an immune mechanism triggered by the metabolite trifluoroacetic acid. However, due to a much lower metabolism by CYP2E1 compared to halothane, sevoflurane is generally not associated with immune-related hepatic toxicity and is widely used, even in liver surgery [12].

The protective mechanisms of sevoflurane against HIRI are complex and involve multiple pathophysiological pathways. Over the past decades, numerous studies have been published reporting such protective effects of sevoflurane and attempting to elucidate its underlying mechanisms. Initially, most of these studies were conducted in the heart, but similar effects were later described in other organs, such as the lungs, brain and liver [13,14,15]. Several pathophysiological mechanisms have been identified, including reduction of oxidative stress, increased expression of anti-apoptotic factors, inhibition of the NF-κB (Nuclear factor kappa-light-chain-enhancer of activated B cells) signaling pathway, reduced formation or prevention of opening of the mPTP (mitochondrial permeability transition pore), protection of the glycocalyx, upregulation of hypoxia inducible factors (HIFs) and modulation of innate and adaptative immunity [1,2].

While multiple clinical studies showed a clinical benefit of sevoflurane administration during coronary artery bypass graft surgery [16,17], its benefit in liver surgery and transplantation remains controversial. 

The aim of this scoping review is to systematically map the available literature studying the potential benefits of sevoflurane on HIRI and to identify any existing gaps in knowledge. In order to assess the clinical significance of sevoflurane protection, as well as its underlying mechanism, both experimental and clinical studies were included.

## 2. Methods

This scoping review was conducted using the Joanna Briggs Institute (JBI) framework for scoping reviews [18]. 

### 2.1. Search Strategy

A preliminary search for relevant documents was performed on the databases Medline and Embase. The text words contained in the titles and abstracts of relevant articles, as well as the index terms linked to those articles, were then used to develop a full search strategy for Medline and Embase up to February 2022. The reference lists of the selected sources were then screened for other relevant articles. The full search protocol can be found in the Appendix A.

### 2.2. Inclusion Criteria

In order to assess the clinical significance of sevoflurane pre- and postconditioning on HIRI, as well as its underlying mechanisms, both animal and clinical studies were included for analysis. Experimental models of HIRI, studies in the setting of liver resections with portal triad clamping and LT were considered for inclusion. Only publications about the VA sevoflurane were included. All types of study designs were included, with no restriction regarding time of publication. Conference abstracts, case reports, qualitative reviews and opinion articles were excluded. For feasibility reasons, only articles published in the English language were included.

### 2.3. Study Selection

Following the search, all selected references were uploaded to EndNote and duplicates removed. Reference titles and abstracts were screened independently by two reviewers. After screening, 50 potentially relevant sources were assessed in full against the inclusion criteria, and a total of 45 studies were included. Discrepancies between both reviewers were resolved through discussion until full agreement was reached.

**Figure 1:**
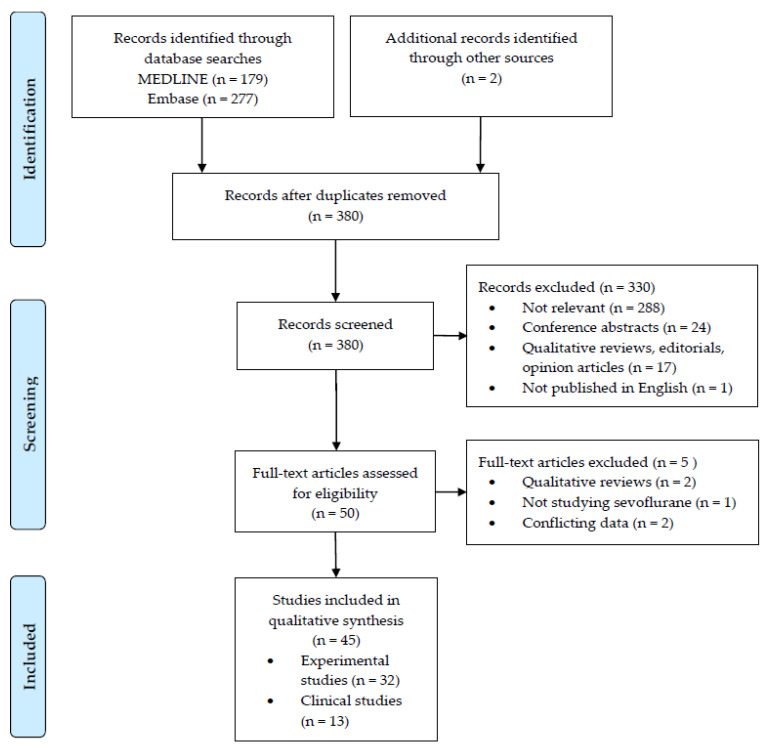
Study Flow Chart.

A flow diagram of the study review process, in accordance with the Preferred Reporting Items for Systematic Reviews and Meta-analyses extension for scoping reviews (PRISMA-ScR) [19], can be found in Figure 1.

### 2.4. Data Extraction

Relevant data were extracted from the selected sources by the reviewers, using a predefined data extraction form including details about the publication type, year of publication, population, context, methods and key findings of the selected sources. Data were repeatedly updated throughout the reviewing process.

## 3. Results

### 3.1. Experimental Studies

Our research led to the identification of 32 articles using different experimental models: 22 employed a rodent model, 6 were performed using a murine model, 1 study was performed with rabbits, only 2 studies used a large animal model (pigs) and 1 study utilized an in vitro model.

At the cellular level, hepatocyte death is the result of all the molecular reactions that take place during the HIRI phenomenon. In most experimental studies, sequential serum measurements of alanine aminotransaminase (ALT), aspartate aminotransaminase (AST) and lactate dehydrogenase (LDH) levels were used as surrogate biomarkers to quantify the intensity and progression of hepatocyte death during HIRI.

In an overwhelming majority, the authors concluded sevoflurane has a protective effect on HIRI. Only Bellanti et al. showed, in a rodent model of 70% hepatic ischemia of 45 min followed by 60 min of reperfusion, a superiority of intravenous propofol conditioning over sevoflurane conditioning through better mitochondrial preservation and reduced AST, ALT and reactive oxygen species (ROS) production. Inhibition of hypoxia-inducible factor 1 alpha (HIF-1α) by propofol was the molecular mechanism proposed by this team to explain this phenomenon [20]. Seven studies compared sevoflurane to isoflurane or halothane [21,22,23,24,25,26,27], five of them concluding the superiority of sevoflurane [22,23,24,26,27]. In 2008, Bedirli et al. demonstrated the superiority of sevoflurane over isoflurane preconditioning by the reduction of ALT, AST and malondialdehyde (MDA), a marker of oxidative stress, and the increase of hepatic tissue blood flow in the sevoflurane group [22]. In 2011, Soubhia et al. reported that, in a rodent model of phenobarbital-induced liver injury, animals subjected to hypoxia plus sevoflurane conditioning showed fewer microscopic liver alterations (steatosis, inflammatory infiltration, or necrosis) compared to the halothane-conditioned group [23]. A decade later, Yang et al. concluded that sevoflurane was superior when compared to isoflurane conditioning after the results indicated all biomarkers of HIRI were significantly lower in the sevoflurane group [26].

Only two studies compared different protocols of sevoflurane administration. Shiraishi et al. found HIRI intensity to be similar in a preconditioning protocol compared with a postconditioning protocol [28]. Figueira et al. did not show statistical differences for transaminases and lactate levels between a preconditioning group compared to a pre- plus postconditioning group. However, interleukin 6 (IL-6) levels were decreased when a pre- and postconditioning strategy were combined [29].

Regarding the concentration of sevoflurane, Zhou et al. used a preconditioning protocol in rats with 3 different concentrations of sevoflurane (2.4%, 3.6% and 4.8%). The authors found no dose–response relationship between sevoflurane preconditioning and its protective effect against HIRI. ALT, AST, myeloperoxidase (MPO) and MDA were reduced and superoxide dismutase (SOD) increased in all preconditioned groups when compared to the control groups, but there were no statistical differences between the groups with different concentrations of sevoflurane [30].

In addition to the pharmacological intervention with VAs, IPC, which consists of the application of a short period of ischemia (about 10 min) and reperfusion before sustained ischemia, can be used in order to limit HIRI. Morita et al. showed no differences in terms of HIRI intensity between a sevoflurane-preconditioned group and an IPC group [31]. Similarly, Balzan et al., in one of only two studies using a porcine model of HIRI, found no differences in serum levels of AST, ALT, amylase, alkaline phosphatase (ALP) and total and direct bilirubin between the sevoflurane preconditioning group and the IPC group. However, compared to the control group, only the sevoflurane preconditioning group had significantly less DNA damage after reperfusion [32]. In a study performed in rats comparing the effects between the association of an IPC protocol with sevoflurane or isoflurane conditioning and pharmacological conditioning alone, Jeong et al. were able to show an additional protection of IPC when VA conditioning was used [25]. Conversely, Yamada et al., in the only study using rabbits, found that the addition of an IPC period to sevoflurane conditioning did not result in more protection against HIRI compared to sevoflurane conditioning alone [33].

Many of these experimental studies give insight into the molecular mechanisms associated with HIRI. Several mechanisms have been proposed, including the reduction of inflammatory cytokine secretion, reduction of oxidative stress, downregulation of apoptotic pathways and reduction of complement activation. 

An inflammatory cytokine storm is mainly caused by the secretion of several cytokines involved in inflammation; ischemia-reperfusion injury, therefore, is sometimes characterized as “sterile inflammation”. These cytokines are mainly IL-1, IL-6 and tumor necrosis factor alpha (TNF-α), whose release is reduced by the administration of sevoflurane in many studies [26,34,35,36,37,38,39,40,41,42,43,44]. One of the most accepted mechanisms associated with the downregulation of such proinflammatory cytokines secretion is the inhibition of the NF-κB pathway, a key regulator of the proinflammatory cytokine pathway. 

Recently, mechanisms associated with the sevoflurane-induced downregulation of inflammation have been further defined. They are likely to be mediated by microRNAs (miRNA and miR), single-stranded non-coding RNA (21-23 nucleotides) playing a role in post-transcriptional regulation of gene expression. It is now well established that miRNA play a central role in regulating multiple pathophysiological pathways associated with IRI [45,46]. In 2019, Liao demonstrated that sevoflurane conditioning overexpressed miR-9-5p, targeting the NF-κB3 gene (coding for transcription factor p65), resulting in a reduction of nuclear p65 and, in fine, NF-κB activity [38].

It has been shown that sevoflurane suppresses IRI-induced phosphorylation of NF-κB subunit p65, thus further reducing NF-κB activity [35,44]. In 2021, Xu et al. demonstrated an upregulation of miR-142 with sevoflurane administration, resulting in decreased high mobility group box 1 (HMGB1) expression. HMGB1 is a protein that acts as a danger-associated molecular pattern (DAMP) and is known to activate the NF-κB pathway after binding to toll-like receptor 4 (TLR4) [42].

Still in the field of reduction of this sterile inflammation, several other studies have demonstrated that sevoflurane inhibits leucocyte recruitment in the liver, as shown by reduced hepatic MPO [30,44,47]. At the molecular level, other studies showed that sevoflurane reduces intercellular adhesion molecule 1 (ICAM1) mRNA or ICAM1 expression (an integrin responsible for leucocyte migration) [26,48]. Xu et al. demonstrated a reduction in metalloproteinase-9 (MMP-9) activity with sevoflurane treatment. MMP-9 are involved in the degradation of the extracellular matrix and are known to play a role in leucocyte migration [47].

Furthermore, sevoflurane has been associated with a reduction of plasmatic levels of complement component 3 (C3), and by consequence, with the inhibition of complement activation [26,48].

One of the most important mechanisms of cell death associated with HIRI is apoptosis. Its intensity is commonly quantified by TUNEL assay, which measures the level of DNA fragmentation. Eleven studies demonstrated a reduction of the apoptotic rate in sevoflurane-treated animals [25,31,35,36,37,39,41,43,49,50,51]. The main mechanism explaining this is a reduction of IRI-induced upregulation of proapoptotic proteins, such as Bak or Bax, and an enhancement of anti-apoptotic proteins of the B-cell lymphoma 2 (Bcl-2) proteins family, resulting in an antiapoptotic balance [25,35,50,51].

In 2015, Morita et al. identified four microRNAs suppressed by sevoflurane administration, resulting in an activation of the Akt-glycogen synthase kinase (GSK)-cyclin D pathway, favoring hepatocellular proliferation and inhibiting apoptosis [31]. In 2018, Liu et al. highlighted the role of sevoflurane in inhibiting glucose regulatory protein 78 (Grp78), a key protein that regulates pro-apoptotic pathways (PERK, eIF2-alpha and p-c-JNK/JNK) [36]. Moreover, Sima et al. showed a reduction of apoptosis with sevoflurane through the activation of the JAK2-STAT3 pathway and the inhibition of mPTP [37]. 

In addition, Wu et al. showed that sevoflurane downregulated miR-200, resulting in a reduction of ZEB-1 gene expression, which is involved in H_2_O_2_-dependent apoptosis [49]. Furthermore, the concentration of miR-218-5p decreases with the administration of sevoflurane, leading to GAB2 overexpression. GAB2 is a positive regulator of the phosphatidylinositol 3-kinase (PI3K)-AKT- mechanistic target of rapamycin (mTOR) pathway, which is involved in cell apoptosis [43]. Upstream, Xiao et al. reported that sevoflurane inhalation was associated with the activation of the hepatocyte growth factor (HGF)/Met-tyrosine kinase receptor (MET) pathway [41]. MET is known to activate several pathways involved in tissue regeneration, including the PI3K/AKT and the STAT pathways [52]. 

According to He et al., sevoflurane promotes miR-96 expression, which results in a reduction of FOXO4 expression, which, in turn, leads to overexpression of Bcl-2 and decreased expression of caspase 3, a key mediator of apoptotic cell death, and Bax [50].

One of the most important strategies to alleviate HIRI is to reduce oxidative stress. Administration of sevoflurane has been associated with a reduction of MDA and with an increase of SOD levels, marking a reduction of cellular oxidative stress [22,26,30,35,36,39,41,42,44,47,49,51]. In 2021, Ma et al. demonstrated that sevoflurane preconditioning led to an activation of the Nrf2/Heme-oxygenase-1 (HO-1) pathway by showing that the HIRI protective effect of sevoflurane was counteracted by administration of ML385, an inhibitor of Nrf2. It is commonly admitted that this pathway is involved in mitochondrial oxidative stress [39]. In addition, Shiraishi showed that sevoflurane induces the activation of HO-1, which is associated with reduction of HIRI [28]. In an in vitro study performed using liver tissue biopsies from patients submitted to the Pringle maneuver, Beck-Schimmer et al. established that hepatic stellate cells may play an important role in sevoflurane protection by attenuating the production of ROS, thereby protecting hepatocytes from apoptosis [51].

It is now well recognized that mitochondria play a central role in ROS production and in the initiation of processes leading to necrosis and apoptosis associated with IRI. During IRI, alterations of the mitochondrial electron transport chain lead to the formation of ROS that initiate multiple pathways causing tissue injury. ROS can cause direct damage by altering mitochondrial or cellular lipids and proteins. High levels of ROS, as well as ATP depletion and dysregulation of calcium levels seen during ischemia, act as triggers for mPTP assembly and opening [53,54]. Uncontrolled opening of mPTP results in the release of substances like cytochrome C, succinate and mitochondrial DNA into the cytosol, which act as pro-apoptotic messengers. They can also act as danger-associated molecular patterns (DAMPs), activating innate immunity and systemic inflammatory response.

Experimental data from myocardial IRI models show that sevoflurane protection could be, in fact, partially induced by low levels of the ROS superoxide resulting from a sevoflurane-mediated attenuation of the mitochondrial electron transport chain [2,53]. Superoxide indirectly causes opening of mitochondrial K^+^-ATP and an influx of K^+^. The minor reduction in membrane potential is believed to reduce mPTP assembly. Additionally, superoxide prevents the opening of mPTP through the reperfusion injury salvage kinase (RISK) pathway, a group of protein kinases promoting cell survival and including PI3K/Akt and the downstream target glycogen synthase kinase 3 β (GSK3β). The survivor activating factor enhancement (SAFE) pathway, involving the activation of JAK and STAT3, also plays a role [53]. If most experimental studies investigating the role of the mitochondria in IRI were performed in the heart, the mechanisms would be believed to be similar in other organs.

Additionally, Li et al. showed a protection of endothelial glycocalyx with sevoflurane, as shown by a reduced release of heparan sulfate and syndecan-1 in that group [55]. Glycocalyx plays a central role in endothelial homeostasis and its degradation in the case of IRI leads to increased vascular permeability, oedema, platelet aggregation, hypercoagulability and inflammation [56]. 

At last, Granja et al., studying the molecular pathway transducing the sevoflurane signal itself, revealed that the transduction was mediated by adenosine A_2B_ (Adora2b) receptors. Indeed, the hepatoprotective effect of sevoflurane was abolished in knock-out mice for this receptor [40].

**Figure 2:**
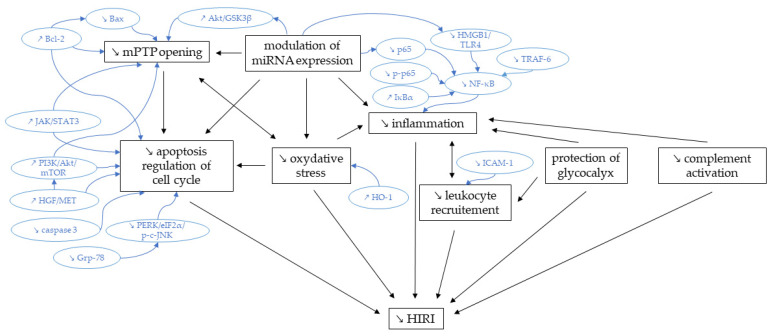
Biological processes involved in sevoflurane protection against HIRI. Akt = protein kinase B; Bax = Bcl-2-associated X protein; Bcl-2 = B-cell lymphoma 2; eIF2α = Eukaryotic Initiation Factor 2 α; Grp-78 = glucose-regulated protein 78; GSK3β = glycogen synthase kinase-3 beta; HGF = hepatocyte growth factor; HIRI = hepatic ischemia-reperfusion injury; HMGB1 = high mobility group box 1; HO-1 = heme oxygenase 1; ICAM-1 = Intercellular Adhesion Molecule 1; IκBα = NF-κB inhibitor alpha; JAK = janus kinase; JNK = c-Jun N-terminal kinase; MET = Met tyrosine kinase receptor; miRNA = microRNA; mPTP = mitochondrial permeability transition pore; mTOR = mechanistic target of rapamycin; NF-κB = Nuclear factor kappa-light-chain-enhancer of activated B cells; PERK = protein kinase R-like endoplasmic reticulum kinase; PI3K = Phosphoinositide 3-kinase; p-p65 = phosphorylated p65; STAT3 = signal transducer and activator of transcription protein 3; TLR4 = toll-like receptor 4; and TRAF-6 = TNF receptor-associated factor 6.

Figure 2 gives an overview of the proposed mechanisms of sevoflurane protection against HIRI. 

A summary of the included experimental studies can be found in Table 1.

### 3.2. Clinical Studies

#### 3.2.1. Liver Resections

In 2008, Beck-Schimmer et al. studied several biomarkers of liver injury and the incidence of post-operative complications after hepatectomies with inflow occlusion [59]. Sixty-four patients were randomized into an intervention group, where propofol was replaced by sevoflurane for 30 min prior to vascular clamping, or into a control group without sevoflurane preconditioning. Both peak transaminases and the incidence of postoperative complications were significantly reduced in the intervention group. In a subgroup analysis of steatotic patients, preconditioning seemed to offer even better protection. Moreover, the authors showed a significant upregulation of inducible nitric oxide synthase (iNOS) mRNA upon reperfusion in the preconditioning group, suggesting that the protective effects of sevoflurane may be mediated by nitric oxide (NO). In a 3-arm randomized controlled trial (RCT) published in 2012 [58], the same authors compared the effect of sevoflurane postconditioning (i.e., discontinuation of propofol and administration of sevoflurane for 30 min after reperfusion) with IC and a control group (i.e., continuous clamping without a protective intervention) in the setting of liver resection with inflow occlusion. They showed a significant reduction of peak AST with IC and sevoflurane postconditioning compared to the control group. The hospital length of stay (LOS) and overall complications were also significantly reduced with both protective strategies compared to the control group. A cost analysis based on these two RCTs [60] later suggested a reduction of hospital costs with pre- and postconditioning compared with the control group, however, without reaching statistical significance.

In another RCT, Song et al. [61] compared postoperative liver function after hepatectomy between sevoflurane and propofol anesthesia. The group receiving sevoflurane for maintenance of anesthesia had slightly lower peak transaminase levels compared to the propofol group, but these results were not significant. Other assessed liver function tests and hospital LOS were not different between the groups. 

In a retrospective study published in 2012 by Slankamenac et al. [62], maintenance of anesthesia with sevoflurane did not seem to offer protection against liver injury after hepatectomy with inflow occlusion compared to continuous propofol administration. Indeed, no statistically significant differences in peak transaminases, peak bilirubin, peak creatinine, postoperative complications, 30-days mortality, intensive care unit (ICU) or hospital LOS were detected between the groups. As the choice of the hypnotic agent was left to the discretion of the attending anesthesiologist, the authors draw attention to the fact that sevoflurane might have been used preferentially in patients with more severe comorbidities, thus introducing a potential negative selection bias. 

In a network meta-analysis comparing various protective strategies against HIRI [63], sevoflurane was found to reduce serious adverse events compared to hepatectomy without protective strategy. However, it is important to note that this network meta-analysis published in 2016 only included both clinical trials published by Beck-Schimmer et al. [58,59] and omitted Song’s results.

One publication studied the effect of sevoflurane preconditioning when IC was performed. Rodriguez et al. [64] found no benefit of sevoflurane preconditioning or IPC when IC was used. Interestingly, patients with underlying liver disease were excluded from this RCT. 

#### 3.2.2. Liver Transplantation

Several clinical studies investigated the impact of sevoflurane on IRI in the setting of LT. Minou et al. [65] randomized 60 deceased brain donors (DBD) to receive either sevoflurane or no VA during organ procurement. Peak levels of ALT and AST were lower in the recipients of organs harvested from the sevoflurane group, but the difference was only significant for the peak level of AST. The incidence of early allograft dysfunction (EAD) was significantly lower in the sevoflurane group (16.7% vs 50%, *p* = 0.013). Interestingly, in a subgroup analysis, sevoflurane did not reduce peak transaminases nor the incidence of EAD when only livers without macrovesicular steatosis were considered. In this study, all recipients were anesthetized with sevoflurane.

Similarly, in a retrospective study published in 2018 [66], Perez-Protto et al. investigated the impact of deceased donor exposure to VAs on graft survival (at 30 days and 5 years) after transplantation. There were no significant differences between the VA and no-VA groups for any of the organs, including the liver. A secondary analysis comparing the donors receiving sevoflurane with the no-VA group showed the same results. However, as the sample size was relatively small and the rejection rates were low in both groups, this study may have been underpowered. It should also be noted that no information about the recipients’ anesthetic regimen is provided.

In a large, retrospective, monocentric study on 1291 LT recipients [67], the authors found no benefit of continuous sevoflurane administration to the recipient compared to desflurane or isoflurane. All three anesthetic agents had similar rates of EAD and renal dysfunction. However, the authors noted a non-significant increase in postoperative ALT in the isoflurane group compared to the other groups, suggesting a greater degree of liver injury when this volatile agent was used. Overall, desflurane had the lowest increase in post-transplant ALT and bilirubin, but without reaching statistical significance. Graft survival, hospital LOS and patient survival were similar among the groups. Importantly, the groups differed significantly in regard to warm and cold ischemia time, which were prolonged in the isoflurane group. In a subgroup analysis for high-risk LT like steatosis > 10%, donor age > 60 years or DCD donors, peak ALT were also not significantly different. 

In a RCT comparing sevoflurane and desflurane anesthesia in 62 recipients of living donor LT (LDLT) [68], the authors showed a significant decrease in the incidence of postreperfusion syndrome in the sevoflurane group. Postoperative clinical outcomes, e.g., hospital LOS or acute kidney injury (AKI), were not statistically different between the groups. Postoperative laboratory results, including bilirubin and transaminases, were also not different between the groups.

In a multicentric RCT, Beck-Schimmer et al. [69] randomized 98 recipients of cadaveric liver grafts to receive either sevoflurane or propofol anesthesia. Major complication rates and in-hospital mortality were lower in the sevoflurane group, but without statistical difference. No differences with regard to any of the studied biochemical or other clinical endpoints were observed. 

One clinical trial has compared the effects of sevoflurane and propofol in the setting of pediatric LT [70]. In this RCT, the children receiving sevoflurane for maintenance of anesthesia during LT had a significantly lower incidence of AKI compared to the group receiving a continuous infusion of propofol. Inflammatory markers IL-18 and TNF-α after reperfusion were also significantly reduced in the sevoflurane group. Markers of oxidative stress (SOD, MDA and H_2_O_2_) and IL-10 were not different between the groups. Interestingly, the anesthetic regimen of the donors was not specified by the authors.

A summary of the included clinical studies can be found in Table 2.

## 4. Discussion

The vast majority of the included experimental studies demonstrated an hepatoprotective effect of sevoflurane against HIRI, as shown by a reduction of various biomarkers of liver injury or oxidative stress. In the only study investigating different concentrations of sevoflurane, the authors did not find a dose–response relationship [30]. However, a threshold effect could be present, as previously demonstrated by Obal et al. in a rat heart model, noting that preconditioning with sevoflurane at 1.0 minimum alveolar concentration (MAC) offered better protection than 0.75 MAC, but that there was no additional benefit to increasing the dose above 1.0 MAC [71]. To our knowledge, this threshold effect has not been demonstrated in the specific setting of HIRI. 

Preconditioning, conditioning and postconditioning strategies have been shown to be protective in multiple animal studies. Thus, no conclusions as to the optimal time or duration of sevoflurane administration can be drawn from the experimental data. 

The protective effects of sevoflurane appear to be mediated by not one, but multiple molecular targets. Over the past decade, many animal and in vitro studies have tried to define the processes underlying the hepatoprotective effects of sevoflurane. These include the reduction of oxidative stress, the prevention of mPTP opening and apoptosis, the limitation of pro-inflammatory cytokine release through post-transcriptional regulation mediated by miRNA and the inhibition of leucocyte migration via the reduction of integrin and metalloproteinase expression. The limitation of complement activation and of endothelial glycocalyx degradation also play a role.

However, our review of clinical trials shows conflicting results. In the setting of liver resection surgery, Beck-Schimmer et al. [59] showed a significant reduction of peak transaminases and of postoperative complications with sevoflurane preconditioning. Continuous administration of sevoflurane did not show any significant advantage when compared to propofol in two selected studies [61,62]. However, these two studies have limitations due to their small sample size [61] and retrospective design [62]. 

Put together, these results could indicate that there is a benefit of discontinuous administration of sevoflurane and that its protective effects are linked not only to the timing, but also to the duration of its administration. However, more studies are warranted to define the optimal time and duration of sevoflurane treatment.

When compared to IPC and IC, sevoflurane preconditioning [64] and postconditioning [58] were equivalent regarding postoperative liver injury and clinical outcomes. Furthermore, the association of multiple IRI-mitigating strategies did not seem to offer any additional benefit compared to one strategy alone [64]. 

In the setting of LT, clinical trials have also shown conflicting results. Minou et al. [65] showed a reduction of postoperative peak transaminases and EAD when liver grafts harvested from DBD donors were pre- and postconditioned with sevoflurane. When administered in the recipient (postconditioning) of a living donor liver graft, Li et al. [70] showed a significant reduction in postoperative AKI and in the release of inflammatory markers after reperfusion. However, after deceased donor LT, no benefit of sevoflurane postconditioning was found in other studies [69]. Again, a possible explanation for these discrepancies could be that the timing of sevoflurane administration plays a significant role and that postconditioning alone does not offer the same protective effect as preconditioning or the association of pre- and postconditioning. Two retrospective studies investigated long-term graft survival (up to one and five years) with sevoflurane post- and preconditioning, respectively [66,67]. In these studies, the authors could not demonstrate a long-term benefit of sevoflurane treatment. Surprisingly, in a small RCT studying the effects of sevoflurane in living donor kidney transplantation, Nieuwenhuijs-Moeke et al. found a significantly lower T cell-mediated rejection rate after two years when the grafts were postconditioned with sevoflurane [72]. However, it is unclear whether these results should be seen as a long-term immunological benefit of sevoflurane treatment and if these results can be extrapolated to LT. 

In two RCTs, sevoflurane was found to be particularly beneficial in subjects with macrovesicular steatosis [59,65]. This could be partially explained by the bigger degree of organ injury observed in that subgroup, as steatotic livers are known to be less tolerant of IRI [73]. When comparing subgroups of cirrhotic with non-cirrhotic patients, Song et al. [61] found a non-significant increase in serum transaminases after hepatectomy with inflow occlusion in the cirrhosis group. As the duration of inflow occlusion was relatively short in this study, the authors hypothesize that a longer period of ischemia could have unmasked a more significant difference between the cirrhotic and non-cirrhotic patients. In their retrospective study comparing different VAs in LT recipients, Mangus et al. found no difference in biomarkers of HIRI, even in a subgroup analysis for marginal grafts [67]. More well-designed studies are needed to determine whether sevoflurane exerts its protective effects preferentially on marginal livers.

When looking at clinical outcomes, two trials demonstrated a significant reduction of in-hospital complications after hepatectomy when sevoflurane was used [58,59]. However, none of the selected clinical trials showed a reduction in other important clinical outcomes, such as mortality or ICU stay. This lack of clinically relevant systemic effect could be partially explained by the other protective and supportive measures put in place during and after surgery. Interestingly, propofol, a widely used intravenous hypnotic agent used for induction and maintenance of anesthesia, as well as for continuous sedations in the ICU, has been reported to exert a protective effect on HIRI in multiple experimental studies [35,74]. It is important to note that in most clinical trials, the control group was given propofol for maintenance of anesthesia. As the control groups could have benefitted from the protective effects of propofol, the clinical benefit of sevoflurane might not have been detected. Similarly, the VAs desflurane and isoflurane, used as comparators in several studies, are also known to protect the liver from IRI [25,75,76]. 

It is important to underline the great variability in the degree of ischemic injury observed in the included clinical trials. Indeed, it varies from a short period of programmed vascular clamping in the setting of liver resection surgery to prolonged warm and cold ischemia times in the case of deceased donor LT. These grafts suffer a chain of serious injuries, including donor cause of death, subsequent hemodynamic and endocrinological disturbances, organ procurement surgery, graft preservation, transport and implantation. It is possible that, when exposed to these serious ischemic insults, the beneficial effect of sevoflurane could be insufficient. On the other hand, the injury observed during liver resection surgery or even LDLT might not be severe enough to reveal the HIRI-mitigating effects of sevoflurane. 

Further objective-designed trials are needed to investigate what type of patient could potentially benefit from sevoflurane treatment, with regard to preexisting liver disease, perioperative medications or extent of ischemic injury. As deceased donor LT is associated with a wide range of potential confounding factors, LDLT could serve as an interesting research model because it provides a relatively homogenous donor population and controlled ischemia times. 

## 5. Conclusions

Sevoflurane seems to protect the liver from HIRI in multiple animal and in vitro models. It acts on multiple molecular targets and results in a reduction of leucocyte migration, inflammatory response and oxidative stress. It limits mPTP opening and subsequent apoptosis, reduces complement activation and protects the endothelial glycocalyx. 

However, the clinical relevance of these phenomena remains unclear. While several trials showed a reduction of early postoperative markers of liver injury, the benefit of sevoflurane on postoperative clinical outcomes and long-time graft survival remains to be demonstrated. More well-designed clinical trials are needed to investigate the optimal clinical setting of sevoflurane application. The time and duration of sevoflurane treatment, preexisting liver disease and the extent of ischemic injury most likely play an important role and need to be further investigated.

## Figures and Tables

**Table 1:** Experimental studies.

Author	Year	Population	HIRI Mechanism	Main Results	Suggested Mechanisms	Comments
Rats						
Imai et al. [21]	1996	16 Sprague Dawley rats (anesthetized with PTB): - 4 I/R (control) - 4 I/R + halothane 2.1% conditioning - 4 I/R + isoflurane 2.9% conditioning - 4 I/R + sevo 4.4% conditioning	Liver excision and ex vivo portal perfusion at 0.2 kPa for 15–30 or 60 min and reperfusion at 1.2 kPa for 120 min	LDH decreased in VA groups after reperfusion (*p* < 0.05)	N/A	N/A
Bedirli et al. [22]	2008	72 Wistar rats (anesthetized with ketamine): - 24 I/R (control) - 24 I/R + sevo 2% conditioning - 24 I/R + isoflurane 1.5% conditioning	Partial HPC (left and median lobe) 45 min + 120–240 min reperfusion	ALT, AST, MDA reduced in sevo group compared to I/R and I/R isoflurane group (*p* < 0.05) Hepatic tissue blood flow increased in sevo group compared to I/R and I/R isoflurane group (*p* < 0.05) IL-1, TNF-α: no statistical differences	N/A	N/A
Kong et al. [34]	2010	60 Sprague Dawley rats: - 30 anesthetized with choral hydrate - 30 sevo 1.5–2.5% conditioning	50% size liver transplantation model	TNF- α, IL-6, MPO, NGAL concentration 2 h after reperfusion decreased in sevo group compared to chloral hydrate group (*p* < 0.05) Renal tissue NF-κB activity higher in chloral hydrate group compared to sevo (*p* < 0.05) No statistical differences for ALT and AST	N/A	NGAL = early predictive biomarker of AKI Sevo conditioning attenuates kidney injury
Soubhia et al. [23]	2011	30 Wistar rats: - 6 control group - 6 Phenobarbital group - 6 Hypoxia group: 120 min at 14% O_2_ - 6 Halothane group: 120 min at 14% O_2_ + halothane 1% conditioning - 6 Sevo group: 120 min at 14% O_2_ + sevo 2% conditioning	Liver hypoxia through ventilation at 14% O_2_–86% N_2_ during 120 min	Significantly less optical microscopic liver alteration (steatosis, inflammatory infiltration, necrosis) compared to halothane group No statistical differences between halothane and sevo regarding AST and ALT	N/A	N/A
Zhou et al. [30]	2013	50 Sprague Dawley rats (anesthetized with PTB): - 10 sham - 10 I/R - 10 I/R + sevo 2.4% preC 30 min - 10 I/R + sevo 3.6% preC 30 min - 10 I/R + sevo 4.8% preC 30 min	Partial HPC (left + median lobe) 60 min + 120 min reperfusion	AST, ALT, MPO, MDA reduced in sevo groups (*p* < 0.05) compared to I/R group SOD increased in sevo groups (*p* < 0.05) compared to I/R group No statistical differences between groups with different sevo concentrations	No dose-response relationship between sevo preC and its protective effect against HIRI	N/A
Dal Molin et al. [24]	2014	20 Wistar rats: - 10 rats anesthetized with sevo 2.4–3.5%: 5 donor rats and 5 recipient rats - 10 rats anesthetized with isoflurane 1.5–2%: 5 donor rats and 5 recipient rats	LT model: liver donor rat, cold liver storage for 360 min before reimplantation in recipient rat	AST, ALT and LDH decreased in preservation liquid of sevo group (*p* < 0.05) No statistical difference in AST, ALT and LDH in serum Serum TBARS concentration decreased in recipient rats in sevo group (*p* < 0.05) NO in liver tissues increased in sevo group (*p* < 0.05)	N/A	TBARS = products of lipid oxidation; markers of OS Serum measurements 15 min after reperfusion and in preservation liquid
Morita et al. [31]	2015	21 Wistar rats (anesthetized with PTB): - 7 I/R (control) - 7 I/R + sevo 2% preC (10 min) - 7 I/R + IPC	Partial HPC (left + median lobe) 60 min + 180 min reperfusion IPC = 10 min clamping + 10 min reperfusion before I/R	ALT, AST decreased in sevo and IPC group compared to control (*p* < 0.05); no statistical differences between sevo and IPC Identification of 4 miRNA suppressed by sevo and IPC; miRNA involved in downregulation of the Akt/GSK/Cyclin D pathway (*p* < 0.05)	Activation of Akt/GSK/cyclin D pathway leading to: - Hepatocellular proliferation - Downregulation of cell apoptosis	N/A
Cavalcante et al. [57]	2015	39 Wistar rats (anesthetized with ketamine + xylazine) - 13 control group - 13 I/R - 13 I/R + sevo 2% conditioning	Partial HPC (left + median lobe) 60 min + 240 min reperfusion	ALT, AST decreased in sevo group (*p* < 0.05) No statistical differences for IL-6, IL-10, TNF- α Preservation of mitochondrial function in sevo group: preserved S3 state respiration, RCR, ADP/O (*p* < 0.05)	Preservation of mitochondrial function	N/A
Mikrou et al. [48]	2016	50 Wistar rats (anesthetized with ketamine + xylazine) - 10 mechanical ventilation only - 10 sham - 10 sham + sevo - 10 I/R - 10 I/R + sevo 1,2% preC (30 min)	Partial HPC (right + median lobe) 45 min + 360 min reperfusion	ALT, ALP, AST, plasmatic C3 and ICAM mRNA decreased in I/R sevo preC group compared to I/R group (*p* < 0.05)	Downregulation of: - ICAM1, leading to reduction of leucocyte recruitment - C3, leading to reduction of complement-induced inflammation	N/A
Li et al. [55]	2016	28 Sprague Dawley rats: - 7 control + ketamine - 7 control + sevo 2% conditioning - 7 I/R + ketamine - 7 I/R + sevo 2% conditioning	Partial HPC (left + median lobe) 45 min + 40 min reperfusion	AST, ALT, HS release, Syn-1 release, microscopic glycocalyx alteration reduced in I/R + sevo group compared to I/R + ketamine group	Protection of endothelial glycocalyx	HS, Syn1 = molecules constituting the glycocalyx
Xu et al. [35]	2016	Sprague Dawley rats (anesthetized with PTB) (>5 per group): - sham - I/R - I/R + propofol - I/R + sevoflurane 3% conditioning	Partial HPC (left + median lobe) 60 min + 120 min reperfusion	AST, ALT, IL-1, IL-6, TNF-α, NO, MDA, Bax, Bak reduced in propofol and sevo group compared to I/R group (*p* < 0.05) IL-10, SOD, Bcl-2, Bcl-xl increased in propofol and sevo group (*p* < 0.05) Reduction of p65 phosphorylation in propofol and sevo group Reduction of p38 phosphorylation in sevo group	Inhibition of p65 phosphorylation; downregulation of NF-κB pathway. Regulation of mitochondrial permeability through upregulation of anti-apoptotic and downregulation of pro-apoptotic molecules	Bax, Bak = pro-apoptotic proteins Bcl-2, Bcl-xl = anti-apoptotic proteins
Bellanti et al. [20]	2016	30 Wistar rats: - 10 anesthetized with tiletamine/zolazepam (5 I/R + 5 sham) - 10 anesthetized with propofol (5 I/R + 5 sham) - 10 anesthetized with sevo 2% ((5 I/R + 5 sham)	PM 45 min + 60 min reperfusion	ALT, AST, ROS decreased in propofol group compared to control group (*p* < 0.05) Better preservation of mitochondrial activity in propofol group (*p* < 0.05) No effect of sevo (AST, ALT, ROS, mitochondrial activity) compared to tiletamine/zolazepam	Suggested protective effect of propofol through inhibition of HIF-α	Study showing no protective effect of sevo against HIRI
Jeong et al. [25]	2017	38 rats: - 3 sham + isoflurane 1.5% - 3 sham + sevo 2.5% - 8 I/R + isoflurane 1.5% conditioning - 8 I/R + sevo 2.5% conditioning - 8 I/R + IPC + isoflurane 1.5% conditioning - 8 I/R + IPC + sevo 2.5% conditioning	Partial HPC (left + median lobe) 45 min + 120 min reperfusion IPC = 10 min clamping + 15 min reperfusion + I/R	ALT, AST decreased in IPC groups compared to I/R (*p* < 0.05) (similar effect for isoflurane or sevo) Bcl-2 mRNA expression increased in IPC groups compared to I/R (*p* < 0.05) (similar effect for isoflurane or sevo) Caspase 3 level: no statistical difference in control group vs. sevo groups	Bcl-2 upregulation	Bcl-2 = anti-apoptotic protein
Liu et al. [36]	2018	24 Sprague-Dawley rats (anesthetized with PTB) - 8 sham - 8 I/R - 8 I/R + sevo 2.4% preC (30 min)	Partial HPC (left + median lobe) for 120 min + 120 min reperfusion	IL-1, IL-6, TNF-alpha, MDA, NO, apoptotic rate reduced in sevo group compared to I/R group (*p* < 0.01) SOD, IL-10 increased in sevo group compared to I/R group (*p* < 0.01)	Inhibition of Grp78 expression (involved in apoptotic pathways)	N/A
Sima et al. [37]	2019	40 Sprague Dawley rats (anesthetized with urethane) - 10 sham - 10 I/R - 10 I/R + sevo preC (30 min) - 10 I/R + sevo preC (30 min) + AG490	Partial HPC (left + median lobe) 60 min + 360 min reperfusion	ALT, AST, ALP, IL-1, IL-6, TNF-alpha reduced in sevo group compared to I/R group (*p* < 0.05) Adjunction of AG490 increased ALT, AST, ALP, IL-1, IL-6 and TNF-α levels (*p* < 0.05) STAT2 and JAK3 expression higher in the sevo group compared to I/R group; effect counteracted by adjunction of AG490 (*p* < 0.05)	Activation of the JAK2-STAT3 pathway Inhibition of mPTP opening	AG490 = inhibitor of JAK2-STAT3 pathway
Liao et al. [38]	2019	36 Sprague-Dawley rats (anesthetized with PTB) - 6 sham - 6 I/R - 6 I/R + miR-9-5p mimic - 6 I/R + miR-9-5p antagomir - 6 I/R + sevo 3% conditioning - 6 I/R + sevo 3% conditioning + miR-9-5p antagomir	PM 60 min + 120 min reperfusion	ALT, AST, LDH, IL-1, IL-6, TNF-α reduced in sevo and miR-9-5p mimic groups compared to I/R group (*p* < 0.01) IL-10 increased in sevo and miR-9-5p mimic groups compared to I/R group (*p* < 0.01) Sevoflurane conditioning suppresses the overexpression of transcription factor p65 triggered by I/R	miR-9-5p overexpression; reduction of p65 by inhibition of its coding gene NF-κB3, subsequent reduction of NF-κB activity	
Shiraishi et al. [28]	2019	48 Wistar rats (anesthetized with PTB, propofol, fentanyl) - 8 sham - 8 I/R - 8 I/R + sevo 2.5% preC (30 min) - 8 I/R + sevo 2.5% postC (30 min) - 8 I/R + sevo 2.5% preC (30 min) + Znpp - 8 I/R + sevo 2.5% postC(30 min) + Znpp	Partial HPC (median + left lobe) 60 min + 180 min reperfusion	ALT, AST and LDH: reduced in sevoflurane groups compared to I/R (similar for pre- or postC) (*p* < 0.05) ALT, AST and LDH reduction is less marked with administration of Znpp (*p* < 0.05)	Increase in HO-1 expression	Znpp = HO-1 inhibitor
Figueira et al. [29]	2019	20 Wistar rats (anesthetized with ketamine and xylazine) - 5 sham - 5 I/R - 5 I/R + sevo 2.5% preC (15 min) - 5 I/R + sevo 2.5% preC (15 min) + postC (20 min)	Partial HPC (median + left lobe) 45 min + 240 min reperfusion	ALT decreased in sevo group compared to I/R (similar for preC or pre + postC) (*p* < 0.05) Potassium and HCO_3_^-^ increased in sevo group compared to I/R (*p* < 0.05) IL-6 decreased in sevo group compared to I/R; effect more marked for pre- + postC group (*p* < 0.05)	N/A	N/A
Yang et al. [26]	2019	40 Wistar rats (anesthetized with PTB) - 10 sham - 10 I/R - 10 I/R + sevo 3% conditioning - 10 I/R + isoflurane 2% conditioning	PM for 45 min + 120 min reperfusion	AST, ALT, LDH, TNF-α, IL-1, IL-6, ICAM-1, MDA, NO, C3: reduced in VA conditioned groups compared to I/R (effect more marked for sevo group) (*p* < 0.05) IL-10 and SOD increased in VA conditioned group compared to I/R (effect more marked for sevo group) (*p* < 0.05)	ICAM1 reduction and subsequent decrease in leucocyte recruitment Decrease in complement activation	N/A
Xu et al. [47]	2019	51 Wistar rats (anesthetized with PTB) - 17 sham - 17 I/R - 17 I/R + sevo 2% preC (30 min)	Partial HPC (median + left lobe) 30 min + 60 min reperfusion	AST, ALT, TNF-α, pulmonary MDA, pulmonary MPO, MMP-9 mRNA decreased in sevo group (*p* < 0.05)	Inhibition of MMP-9 secretion	MMP-9 involved in leucocyte recruitment and activation
Ma et al. [39]	2021	32 Sprague Dawley - 8 sham (PTB) - 8 I/R (PTB) - 8 I/R + sevo 2.4% conditioning - 8 sham + sevo 2.4% in vitro incubation of BRL-3A cells with ML385	Partial HPC (median + left lobe) 120 min + 120 min reperfusion	LDH, MDA, IL-1, IL-6, TNF-α, apoptotic rate, liver injury, cytosolic Nrf2 expression decreased in I/R + sevo group compared to I/R group (*p* < 0.01) HO-1 expression, nuclear Nrf2 expression increased in I/R + sevo group compared to I/R group (*p* < 0.01) Protective effect of sevo was counteracted by ML385 treatment	Activation of Nrf2-HO1 pathway	ML385 = Nrf2 inhibitor
Liu et al. [44]	2021	30 Wister rats (anesthetized with PTB) - 10 sham - 10 I/R - 10 I/R + sevo preC (30 min)	Partial HPC (median + left lobe) 120 min + 120 min reperfusion	Pathological liver damage, AST, ALT decreased in sevo group compared to I/R MPO, TNF-α, IL-1, IL-6 decreased in sevo group compared to I/R Increased IκBα expression in sevo group; decreased TRAF6, p-IκBα, and p-p65 expression	Inactivation of the TRAF6- NK-κB pathway	
Mice						
Granja et al. [40]	2016	Mice (anesthetized with PTB) - I/R wild type (control) - I/R wild type + sevo conditioning - I/R + Adora2a ^−/−^ + sevo 2% conditioning - I/R + Adora 2b ^−/−^ + sevo 2% conditioning In vitro administration of liquid sevoflurane to whole blood	PM 30 min + 180 min reperfusion	Platelet activation, leucocyte activation, AST and IL-6 reduced in sevo conditioned group; protective effects of sevo not observed in Adora2b^−/−^ mice (*p* < 0.05) Activation of platelets and interaction of platelets and neutrophils inhibited in vitro	Protective effects mediated through adenosine receptor Adora2b	N/A
Wu et al. [49]	2016	C57BL/6 mice (anesthetized with ketamine) - sham - I/R - I/R + sevo 2% conditioning	PM 30 min + 30 min reperfusion	ALT, AST, LDH, MDA reduced in sevo group compared to I/R (*p* < 0.05) Overexpression of miR-200c significantly inhibits the protective effects of sevo in HIRI	miR-200c downregulation ZEB-1 (target gene of miR-200c) involved in H_2_O_2_-induced apoptosis	N/A
He et al. [50]	2021	190 C57BL/6 mice separated in different groups combining: - Sham or I/R - sevo 2% conditioning or no sevo - miR-96 antagomir or miR-96 antagomir negative control 30 FOXO4 KO mice: - 10 sham - 10 I/R - 10 I/R + miR-96 antagomir	60 min portal vein occlusion + up to 24 h reperfusion	Reduced liver injury, apoptotic cells, FOXO4-positive cells if sevo conditioning (*p* < 0.05) FOXO4 expression increased if transfection of miR-96 antagomir HIRI and cell apoptosis reduced in FOXO4 KO mice	Sevo promotes miR-96 expression which inhibits FOXO4 expression	FOXO4 is a target gene of miR-96 FOX04 is involved in cell apoptosis by upregulating caspase 3 and Bax and downregulating Bcl-2
Xiao et al. [41]	2021	48 C57BL/6 J mice (anesthetized with ketamine and xylazine): - 12 sham - 12 sham + sevo 2.4% preC for 60 min - 12 I/R - 12 I/R + sevo 2.4% preC for 60 min Addition of 3-MA/HGF inhibitor/phosphate-buffered saline	Partial HPC (median + left lobe) 30 min + 360 min reperfusion	ALT, AST, IL-1, MDA, Suzuki score, TNF-α, apoptotic rate reduced in sevo + I/R group compared to I/R group (*p* < 0.05) SOD, IL-10 increased in sevo + I/R group compared to I/R group (*p* < 0.05) Sevo preC activates autophagy Injection of 3-MA / HGF inhibitor abolishes the protective effects of sevo; HGF overexpression strengthens the protective effects of sevo	Activation of HGF/MET-mediated autophagy	3-MA = autophagy inhibitor
Xu et al. [42]	2021	30 BALB/c mice (anesthetized with PTB): - 6 sham - 6 I/R - 6 I/R + sevo 2% postC 120 min - 6 I/R + sevo2% postC 120 min + agomiR-142 - 6 I/R + sevo2% postC 120 min + antagomiR-142	Partial HPC (median + left lobe) 30 min + 120 min reperfusion	AST, ALT, LDH, Suzuki score, IL-1, IL-6, TNF-α, MDA reduced in sevo group compared to I/R (*p* < 0.01) SOD increased in sevo group compared to I/R (*p* < 0.01) Hepatoprotective effects of sevo enhanced by agomiR-124; counteracted by antagomiR-142	Upregulation of miR-142; decreased expression of HMGB1; inhibition of TLR4/NF-κB pathway	N/A
Ji et al. [43]	2022	30 BALB/c mice (anesthetized with PTB): - 6 sham - 6 I/R - 6 I/R + sevo 2% conditioning Additional injection of agomiR-218-5p, agomiR-218-5p NC, antagomiR-218-5p and antagomiR-218-5p NC	Partial HPC (median + left lobe) 45 min + 120 reperfusion	AST, ALT, LDH, MDA, IL-1, IL-6, TNF-α, caspase 3 expression reduced in sevo group compared to I/R (*p* < 0.01) SOD, IL- 10 increased in sevo group compared to I/R (*p* < 0.01) Hepatoprotective effects of sevo reversed by agomiR-218-5p injection	Downregulation of miR-218-5p expression leading to overexpression of GAB2	GAB2 = activator PI3K/AKT/mTOR pathway
Pigs						
Ishida et al. [27]	2002	19 pigs (anesthetized with ketamine) - 10 I/R + isoflurane 1.4% conditioning - 9 I/R + sevo 2.1% conditioning	PM 30 min + 240 min reperfusion	No statistical differences in ALT, AST, LDH, α-GST, lipide peroxides Lactatemia lower in sevo group 120 min after reperfusion	N/A	N/A
Balzan et al. [32]	2014	18 swine (anesthetized with ketamine, midazolam and fentanyl): - 6 I/R (control) - 6 I/R + 30 min sevo preC - 6 IPC	I/R = 40 min PM + 40 min reperfusion IPC = 10 min PM + 15 min reperfusion + I/R	AST, ALT, ALP and bilirubinemia: no significant difference between the groups CRP after ischemia lower in sevoflurane group compared to control (*p* < 0.05) Lower DNA damage in sevoflurane group compared to control (*p* < 0.05)	N/A	N/A
Rabbits						
Yamada et al. [33]	2018	36 white rabbits (anesthetized with ketamine + xylazine) - 9 I/R + propofol + buprenorphine - 9 IPC + propofol + buprenorphine - 9 I/R + sevo 2% conditioning - 9 IPC + sevo2% conditioning	Partial HPC (right lobe) 90 min + 180 min reperfusion IPC = 10 min of clamping + 10 min reperfusion + I/R	No statistical difference for ALT, AST between the groups Galactose clearance increased in sevo groups Lactatemia decreased in sevo groups No added benefit of IPC when sevoflurane is used	N/A	N/A
In vitro						
Beck-Schimmer et al. [51]	2018	In vitro examination of liver biopsy samples taken during an RCT [58], 45 min after reperfusion (propofol anesthesia) - IC (control) - PM > 30 min - PM > 30 min and sevo 3.2% postC for 10 min In vitro exposure of hepatocytes and HSC to H/R with or without sevo	H/R model: exposure of HSC or hepatocytes to 0.2% O_2_ + reoxygenation (21% O_2_) for up to 24 h	Reduction of Bax/Bcl2 mRNA ratio in sevo postC group compared to control (*p* < 0.0.5) Reduction of ROS in HSC exposed to sevo (*p* < 0.05) Caspase activation in hepatocytes incubated with supernatants of HSC exposed to H/R Caspase activation significantly reduced in hepatocytes incubated with supernatants of HSC exposed to H/R and sevoflurane	Inhibition of apoptosis Hepatoprotective effects of sevoflurane possibly mediated by HSC	Bcl-2 = anti-apopototic protein Bax = pro-apoptotic protein

ADP/O = ADP/oxygen; α-GST = alpha glutathione S-transferase; AKI = acute kidney injury; ALP = alkaline phosphatase; ALT = alanine transaminase; AST = aspartate transaminase; Bcl-2 = B-cell lymphoma 2; C3 = complement component 3; CRP = C-reactive protein; HIRI = hepatic ischemia-reperfusion injury; HGF = hepatocyte growth factor; HMGB1 = High mobility group box 1; HPC = hepatic pedicle clamping; H/R = hypoxia/reoxygenation; HS = heparan sulfate; HSC = hepatic stellate cell; ICAM = intercellular adhesion molecule; IL-1 = interleukine 1; IL-6 = interleukine 6; IL-10 = interleukine 10; IPC = ischemic pre-conditioning; I/R = ischemia-reperfusion; LDH = lactate dehydrogenase; LT = liver transplantation; MDA = malondialdehyde; MMP-9 = metalloproteinase-9; MPO = myeloperoxidase; NF-κB = Nuclear factor kappa-light-chain-enhancer of activated B cells; NC = negative control; NGAL = neutrophil gelatinase-associated lipocalin; NO = nitric oxide; OS = oxidative stress; PM = Pringle maneuver; preC = pre-conditioning; postC = post-conditioning; PTB = pentobarbital; RCR = respiratory control ratio; sevo = sevoflurane; SOD = superoxide dismutase; Syn-1 = syndecan-1; TBARS = thiobarbituric acid reactive substance; TLR4 = Toll-like receptor 4; TNF-α = tumor necrosis factor alpha; VA = volatile anesthetic; WT = wild type.

**Table 2:** Clinical trials.

Author	Year	Type of Study	Population	Main Results	Comments
**Liver Resection**					
Beck-Schimmer et al. [59]	2008	RCT	Liver resection with inflow occlusion (>30 min); 64 patients (anesthetized with propofol): - 34 control group - 30 sevo preC (30 min up to 3.2%)	Peak transaminases, complication rate, major complications: significantly reduced Hospital, ICU LOS: no statistical difference	Patients with cirrhosis excluded Stronger protective effects in patients with steatosis iNOS significantly upregulated in the preC group
Song et al. [61]	2010	RCT	Liver resection with inflow occlusion 100 patients: - 50 propofol group - 50 sevo conditioning group	Peak transaminases, bilirubin, ALP, hospital LOS: no significant difference	Non-significant increase in peak transaminases in cirrhotic patients
Slankamenac et al. [62]	2012	retrospective	Liver resection with inflow occlusion 227 patients: - 86 propofol group - 141 sevo conditioning	Peak transaminases, hospital LOS, ICU LOS, complication rates: no significant difference	Possible negative selection bias: sevoflurane preferentially used in patients with more severe comorbidities
Beck-Schimmer et al. [58]	2012	RCT	Liver resection 115 patients (anesthetized with propofol): - 17 inflow occlusion (>30 min) (control) - 50 IC - 48 inflow occlusion (>30 min) + sevo postC (30 min up to 3.2%)	Peak AST, complication rates, hospital LOS: significantly reduced with postC and IC compared to control No significant difference between IC and sevo postC	Patients with cirrhosis excluded
Rodriguez et al. [64]	2015	RCT	Liver resection with IC 107 patients (anesthetized with propofol): - 36 IPC (10 min) - 34 Sevo preC (20 min at 1.5 MAC) - 36 IC alone (control)	Postoperative transaminases, bilirubin, INR, histological analysis, complication rates, hospital LOS: no significant difference between the groups	Patients with cirrhosis excluded iNOS 1h after reperfusion similar to baseline in all groups
Simillis et al. [63]	2016	Network meta-analysis	Liver resection with inflow occlusion	Serious adverse events: significantly reduced. Hospital LOS: no significant difference	Includes only two RCTs [58,59]
Eichler et al. [60]	2017	Cost analysis of two RCTs	Liver resection with inflow occlusion 129 patients (anesthetized with propofol): - 78 sevo preC or postC - 51 propofol alone (control)	Nonsignificant reduction of costs with sevo preC or postC compared to control	Based on two RCTs [58,59] Cost reduction due to significant reduction of complication rates in the preC or postC group
**Liver transplantation**					
Minou et al. [65]	2012	RCT	LT; DBD 60 donors: - 30 sevo 2% preC - 30 No VA (control)	Peak transaminases, incidence of EAD: significantly reduced in sevo group	No significant difference in peak transaminases or EAD in subgroup without steatosis Maintenance of anesthesia in the recipient with sevo in both groups
Beck-Schimmer et al. [69]	2015	RCT	LT 98 recipients: - 50 sevo postC (entire procedure) - 48 propofol (control)	Peak transaminases, incidence of EAD, complication rates, ICU LOS, hospital LOS: no significant difference	Nonsignificant difference in severity of complications in favor of sevo postC group
Lee et al. [68]	2016	RCT	Adult LDLT 62 recipients: - 31 sevo postC (entire procedure) - 31 desflurane (control)	Incidence of PRS: significantly reduced in sevo group Postoperative transaminases, bilirubin, hospital and ICU LOS: no significant difference	Estimated blood loss: significantly reduced in sevo group Donor’s anesthetic regimen unknown
Mangus et al. [67]	2018	retrospective	LT 1291 recipients: - 392 sevo postC - 102 desflurane - 797 isoflurane	Incidence of EAD, renal dysfunction, hospital LOS, graft and patient survival: no statistical difference	Nonsignificant increase in ALT in isoflurane group Warm and cold ischemia times significantly higher in isoflurane group MELD and D-MELD significantly higher in sevo group Subgroup analysis for high-risk grafts: no significant difference in peak ALT
Perez-Protto et al. [66]	2018	retrospective	DBD donors 213 organ donors (173 LT): - 138 VA preC (59 sevo preC) - 75 no VA	Early (30 days) and late (5 years) graft survival: no significant difference Secondary analysis comparing sevo preC and no VA group: no significant difference in early and late graft survival	Recipient’s anesthetic regimen unknown
Li et al. [70]	2019	RCT	Pediatric LDLT 120 recipients: - 60 sevo postC - 60 propofol (control)	Incidence of AKI, IL-18, TNF-α, NGAL: significantly reduced in sevo postC group IL-10, markers of oxidative stress: no significant difference	Donor’s anesthetic regimen unknown

AKI = acute kidney injury; ALP = alkaline phosphatase; DBD = donation after brain death; EAD= early allograft dysfunction; IC = intermittent clamping; ICU = intensive care unit; iNOS = inducible nitric oxide synthase; INR = international normalized ratio; IPC = ischemic preconditioning; LDLT = living donor liver transplantation; LOS = length of stay; LT = liver transplantation; MELD = model for end-stage liver disease; NGAL = neutrophil gelatinase-associated lipocalin; preC = preconditioning; postC = postconditioning; PRS = postreperfusion syndrome; RCT = randomized controlled trial; sevo = sevoflurane; VA = volatile anesthetic.

## Appendix A. Search Strategy

PubMed: ((((((((sevoflurane[MeSH Terms]) OR (anesthetics, inhalation[MeSH Terms])) OR (sevoflurane)) OR (inhaled anesthetic)) OR (inhaled anesthesia)) OR (volatile anesthetic)) OR (volatile anesthesia)) AND (((((((((((((((“reperfusion injury”[MeSH Terms]) OR (reperfusion)) OR (preconditioning)) OR (postconditioning)) OR (ischemia reperfusion)) OR (ischemia))) OR (“transplantation conditioning”[MeSH Terms])) OR (transplantation conditioning)) OR (pharmacological conditioning)) OR (anesthetic conditioning)) OR (primary graft dysfunction[MeSH Terms])) OR (primary graft dysfunction)) OR (graft dysfunction)) OR (early allograft dysfunction))) AND (((((((((“liver transplantation”[MeSH Terms]) OR (“hepatectomy”[MeSH Terms])) OR (hepatectomy)) OR (hepatectomies)) OR (liver resection)) OR (liver transplant)) OR (liver transplantation)) OR (hepatic)) OR (liver))

Search performed on 18 February 2022

Embase: (‘sevoflurane’/exp OR sevoflurane OR ‘inhalation anesthetic agent’/exp OR ‘inhalation anesthetic agent’) AND (‘reperfusion injury’/exp OR ‘reperfusion injury’ OR ‘preconditioning’/exp OR preconditioning OR ‘postconditioning’/exp OR postconditioning OR ‘primary graft dysfunction’/exp OR ‘primary graft dysfunction’) AND (‘liver transplantation’/exp OR ‘liver transplantation’ OR ‘liver graft’/exp OR ‘liver graft’ OR ‘liver resection’/exp OR ‘liver resection’ OR ‘liver’/exp OR liver OR ‘liver cell’/exp OR ‘liver cell’)

Search performed on 28 February 2022
